# The collection of five interesting cases of adrenal tumors from one medical center

**DOI:** 10.1186/1477-7819-12-377

**Published:** 2014-12-08

**Authors:** Anna Babinska, Rafał Peksa, Renata Swiątkowska-Stodulska, Krzysztof Sworczak

**Affiliations:** Department of Endocrinology and Internal Medicine, Medical University of Gdansk, Gdansk, Dębinki St, 80-288 Poland; Department of Pathology, Medical University of Gdansk, Gdansk, Dębinki St, 80-288 Poland

**Keywords:** Medullary hyperplasia, Hydatid adrenal disease, Primary adrenal angiosarcoma, Adenomatoid tumor

## Abstract

**Introduction:**

Adrenal tumors are detected incidentally in 4 to 8% of patients in imaging studies. Adenomas, pheochromocytomas and adrenocortical carcinomas represent the most common tumors of the adrenal glands. Rarely are final histopathological reports are surprising.

**Aim:**

The aim of our study is a retrospective analysis of selected clinical characteristics and hormonal studies in five cases of rare adrenal tumors.

**Materials and methods:**

We present five interesting cases of adrenal tumors: two medullary hyperplasia, one adenomatoid tumor, one hydatid cyst and a primary angiosarcoma of the adrenal gland. The final diagnosis was established by means of microscopic examination of the specimens.

**Conclusions:**

The number of adrenal tumors was increased due to widespread use of imaging procedures. In patients without any known extra-adrenal malignancy most lesions are benign, non-hyper functioning adenomas. Adrenal tumors should be evaluated biochemically and radiologically.

## Background

Adrenal tumors are detected incidentally in approximately 4 to 8% of patients in imaging studies [1]. Adenomas, pheochromocytomas and adrenocortical carcinomas represent the most common tumors of the adrenal glands. Most of them are benign tumors but careful evaluation is required to rule out carcinoma and functional adenoma [1, 2]. We present five very rare, interesting cases of adrenal tumors selected from the material of 1,248 patients from the Department of Endocrinology and Internal Diseases, Medical University of Gdansk, Poland.

## Case presentation

### Cases one and two: medullary hyperplasia – AMH (Adrenal medullary hyperplasia)

Two patients were admitted to our hospital in order to examine the cause of muscle weakness headaches, perspiration, palpitation and facial redness. A 46-year-old male presented with hypertension of 15-months duration (case one). The patient’s family had no history of hypertension, cardiac arrhythmias or thyroid disorders.

A 34-year-old female was referred for the evaluation of palpitation and facial redness of one-year’s duration (case two). The patient’s parents and sister had no history of hypertension or other endocrine disorders.

**Figure 1:**
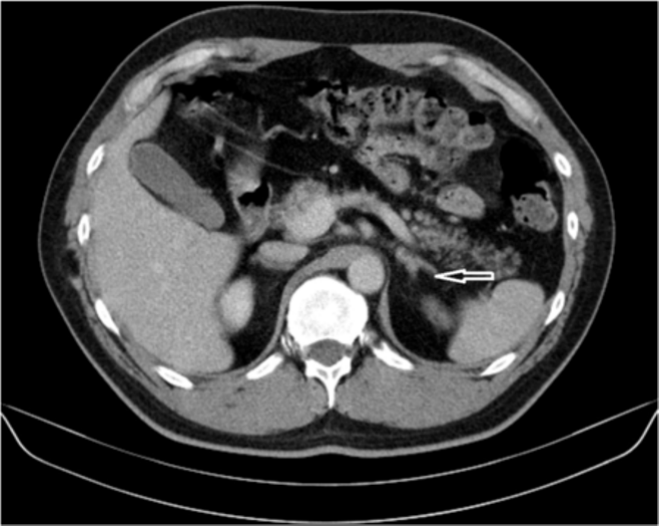
**Computer tomography scan revealed 10 mm left adrenal mass (arrow) – case one.**

**Figure 2:**
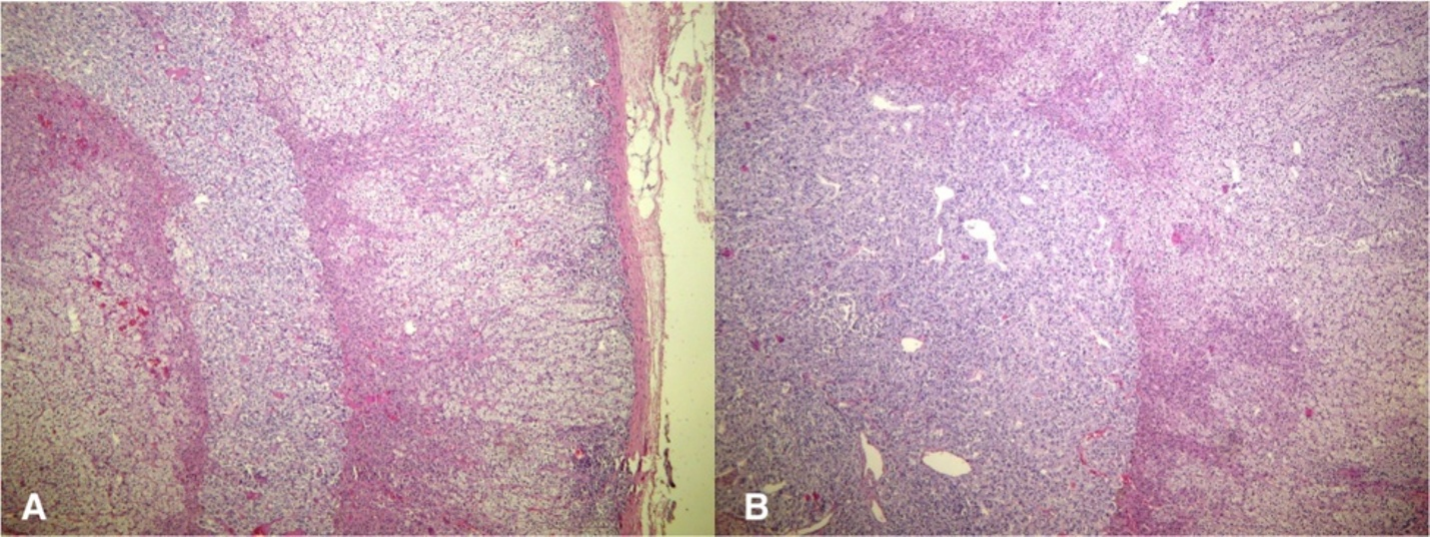
**Diffuse and nodular adrenal medullary hyperplasia.** There is vague nodularity in the medullary compartment **(A)** (H & E, 40× magnification) – case one. There is also diffuse and nodular adrenal medullary hyperplasia and a visible diffuse pattern of hyperplasia **(B)** (H & E, 40× magnification) – case two.

**Figure 3:**
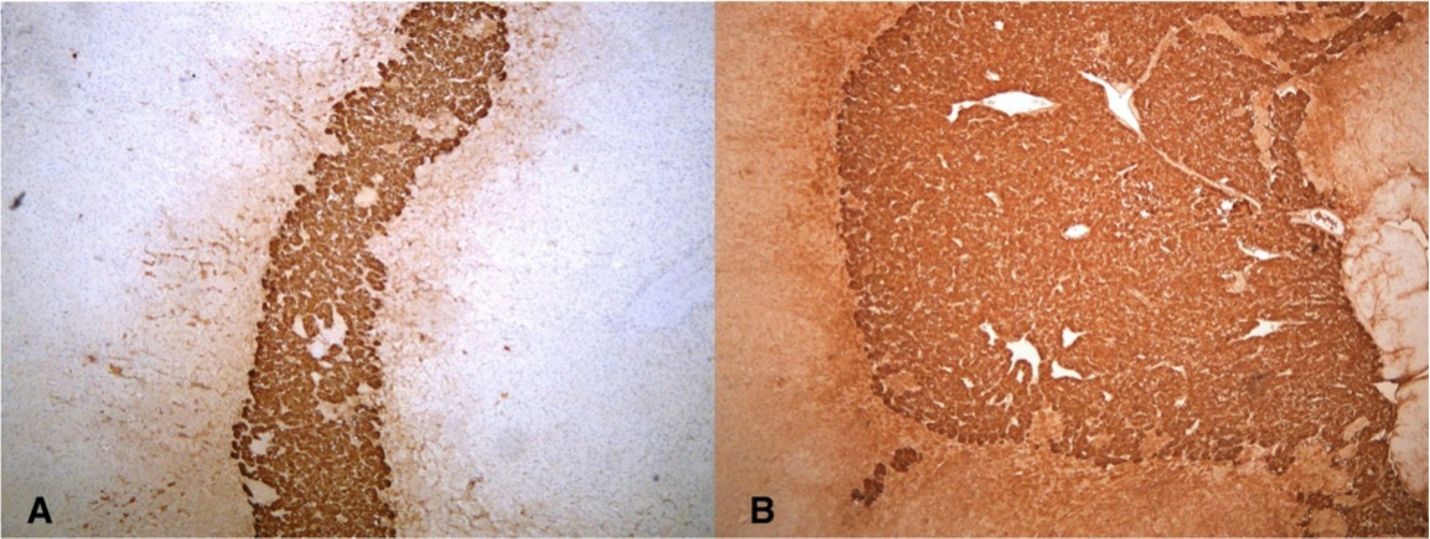
**Adrenal medullary hyperplasia - AMH: chromogranin staining (40× magnification) in case one (A) and case two (B).**

**Figure 4:**
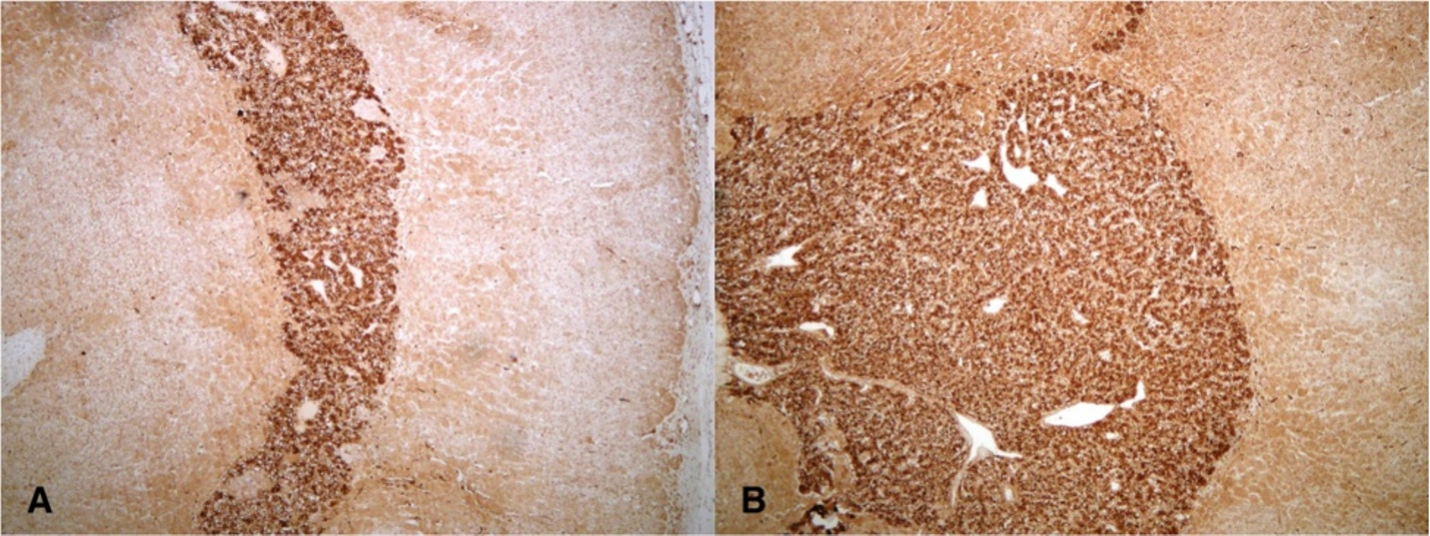
**Adrenal medullary hyperplasia - AMH: S-100 staining (40× magnification) in case one (A) and case two (B).**

On admission, there were no cushingoid signs. Paroxysmal hypertension had occurred, and was poorly controlled by antihypertensive drugs in both cases. Levels of urinary catecholamines were increased during the paroxysmal hypertension (see Table 1). Thyroid scans revealed normal-sized glands and their basic serum calcitonin level was normal. Computer tomography (CT) scans revealed left adrenal masses measuring: 10 mm (patient one - Figure 1) and 30 mm (patient two). Both patients underwent preoperative medical management to block effects of catecholamines released during surgery. The patients underwent laparoscopic total adrenalectomy, with no postoperative complications. Surgery resulted in the normalization of catecholamine hypersecretion and clinical improvement in both patients (Table 1). A light microscopy revealed diffuse enlargement of the adrenal medulla from the head to tail in both cases. The adrenal cortex was normal in both cases. The combined corticomedullary ratio varied between 2:1 (patient one) and 4:1 (patient two), compared with a ratio of 10:1 in a normal gland (Figures 2, 3 and 4). The histopathological examination of the specimens showed diffuse adrenal medullary hyperplasia (AMH).

**Table 1:** **Hormonal characteristic of rare adrenal tumors**

Patient sex/age	Morphology	DHEAS serum	Cortisol urinary	Cortisol serum 8:00 am/8:00 pm	Normetanephtines/metanephrines/VMA urinary	Observation time	Outcome
P1	Normal	80.1	356	290/62	700/550/-	3 years	NED
M/46						Normetanephrines 249	
						Metanephrines 49.1	
P2	Normal	135	215	401.4/68.9	690/442/-	7 years	NED
F/34						Normetanephrines 387	
						Metanephrines 189	
P3	Eosinophilia 2,500/1 mm^3^	33	115	294/54	529/229/-	11 years	NED
F/47						Eosinophilia 350/1 mm^3^	
P4	Anemia	18.15	400	502.4/68.9	-/-/7.35	2 years	Died
F/64	Hb 10.6; Hct 36.3; Mcv 85.4; PLT 309; WBC 9.38						
P5	Normal	425	120	748/132	-/-/5.2	17 years	NED
F/40							

Normal ranges:

serum DHEAS (dehydroepiandrosterone sulphate): 34 to 430 ug/dl;

24-hour cortisol urinary excretion: 12 to 486 nmol/24 hours;

serum cortisol 8:00 am: 101 to 536 nmol/dl;

serum cortisol 8:00 pm: 47 to 458 nmol/dl;

1 mg Dexamethasone suppression test: <50 nmol/l;

24-hour normetanephrines urinary excretion: <600 ug/24 hours;

24-hour metanephrines excretion: <350 ug/24 hours;

24-hour vanilinmandelic acid (VMA): 4 to 8 mg/24 hours;

NED: no evidence of disease.

### Case three: hydatid disease in the adrenal gland

**Figure 5:**
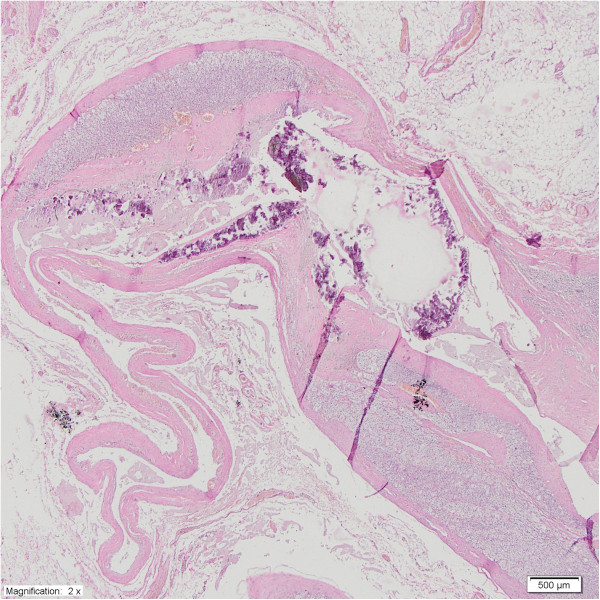
**Case three** - **adrenal cyst.** Calcific deposits are present in the fibrous wall. No lining epithelial or endothelial cells are evident in the cystic space (H & E, 2× magnification).

We present the case of a woman with hydatid disease in her adrenal gland which was found incidentally in an ultrasonography (USG). A 47-year-old woman with two year history of arterial hypertension was referred to our department in 2003. A general examination revealed high blood pressure (180/100 mmHg). No other physical signs suggestive of hypercortisolism were noted. A blood analyses showed hypereosinophilia (see Table 1). A computed tomography (CT) scan of the abdomen demonstrated a sharply marginated, 55 × 58 × 68 mm right cystic mass with internal septa in the right adrenal gland. She underwent a laparoscopic adrenalectomy. In the surgical exploration, a solid cystic mass was defined, compressed against the right kidney’s upper pole. The entire mass, together with normal adrenal tissue, was removed. According to a histopathological examination, hydatid disease in adrenal gland was suspected (Figure 5). Eosinophilia was normalized after surgery (see Table 1). Our patient has continued to be followed up on for 11 years without recurrence of hydatid disease.

### Case four: primary adrenal angiosarcoma

**Figure 6:**
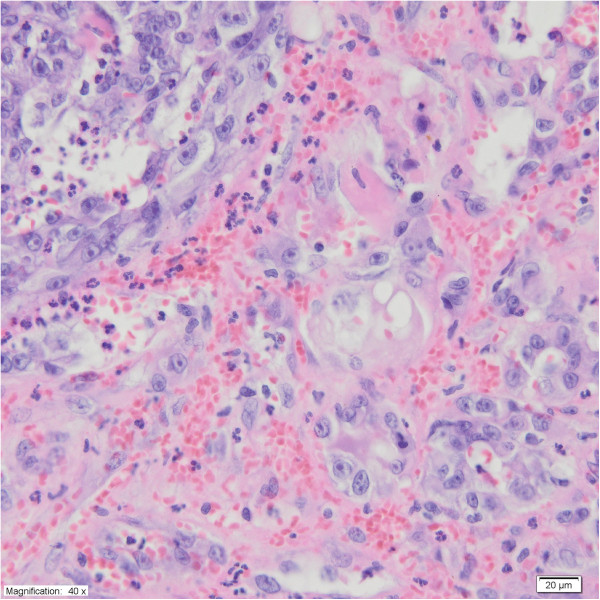
**Case four: angiosarcoma of the adrenal gland is composed of anastomosing vascular channels lined by abnormal endothelial cells that are often pleomorphic, with large hyperchromatic nuclei and prominent nucleoli.** Some of the cells contain cytoplasmic vacuoles and erythrocytes (H & E, 40× magnification).

**Figure 7:**
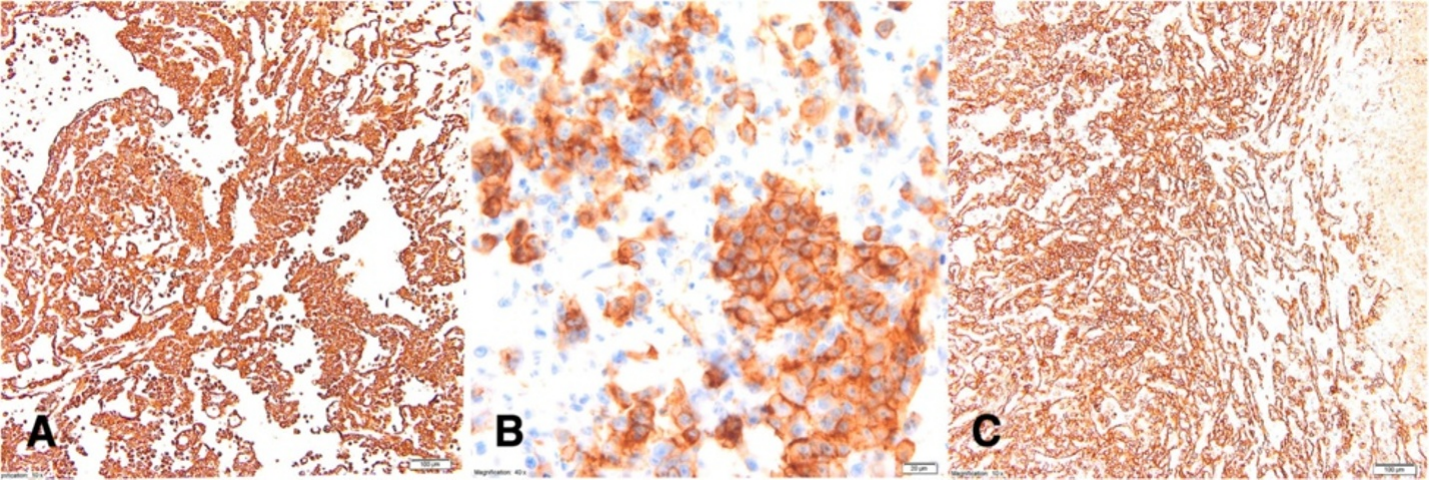
**Case four: Immunohistochemical analysis of the angiosarcoma.** CD31 staining **(A)** – 10x magnification; CD34 staining **(B)**- 40x magnification; vimentin staining **(C)** (10× magnification).

We present the case of a 64-year-old woman with a malignant-looking right adrenal tumor, 87 mm in diameter. She was referred to our department in 2001. She reported a 15 kg weight loss over the previous months and abdominal pain. Her medical history was hypertension and cholelithiasis for over 10 years previously. No other physical signs suggestive of hypercortisolism or pheochromocytoma were noted. A blood analyses showed anemia (see Table 1). As a part of the diagnostic procedures, her clinical examination was followed by an abdominal USG and CT scans. CT scans of the chest and abdomen showed a heterogenous mass of the right adrenal gland, 80 × 78 × 87 mm in diameter, without evidence of local tissue invasion or metastatic spread. Because of suspicion of malignancy, an open adrenalectomy was performed. After mobilization of the splenic flexure, a large mass was identified rising from the right adrenal gland. No obvious lymphadenopathy was seen. The mass was distinct and separate from the surrounding tissues. The entire right adrenal gland containing the tumor was excised. A histopathological examination showed a primary adrenal angiosarcoma (PAA) (Figure 6). Upon immunohistochemical analysis, vascular antigens including CD (cluster of differentiation) 31, CD34 and vimentin, were expressed (Figure 7). Six months after the surgery, a whole body CT scan revealed no other primary tumor site, nor metastases. She died two years later of rapid recurrence of the disease, which metastasized to the bilateral lungs and liver. The chemotherapy plan was doxorubicin and ifosfamide but she refused the treatment.

### Case five: adenomatoid tumor of the adrenal gland

Here we present the case of a 40-year-old woman with an adenomatoid tumor (AT) of the adrenal gland, clinically suspected to be a malignant tumor. The right adrenal tumor was found incidentally on a routine medical examination in 1997. There was no family history of any endocrine pathology and she did not present with any symptoms of palpitations, diaphoresis or flushing. Laboratory examinations revealed that the urinary vanilinmandelic acid, catecholamine and cortisol levels all were within normal limits (see Table 1).

**Figure 8:**
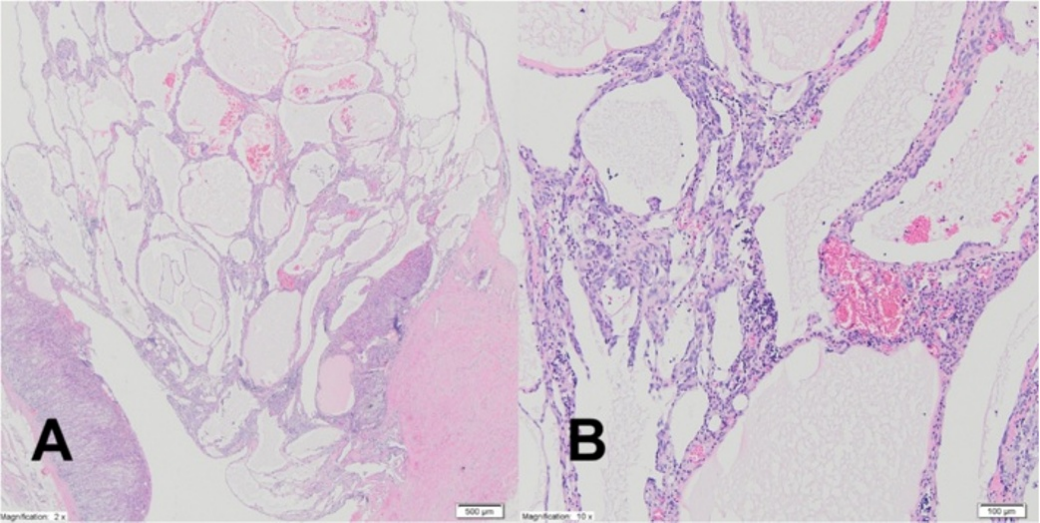
**Case five: mesothelial cells forming dilated, irregular tubules with flattened lining cells which may initially suggest an endothelial orgin.** There is a chronic inflammatory infiltrate in a fibrous stroma **(A)** H & E 2x magnification. At low power, tumor cells form fenestrated channels and anastomosing tubules of varying size, in the left corner there is residue normal adrenal gland parenchyme **(B)** H & E 10x magnification.

**Figure 9:**
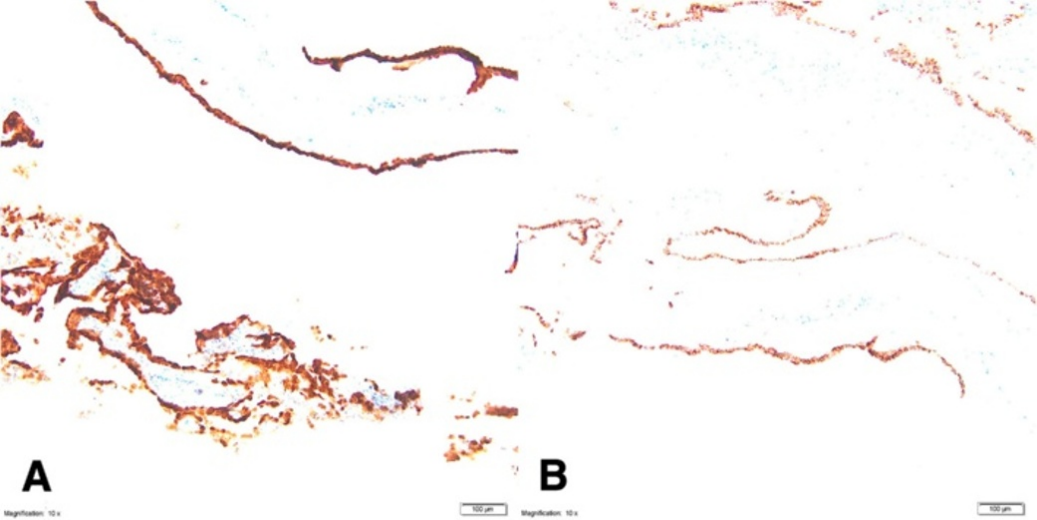
**Case five: adenomatoid tumor cells show positive immunostaining for markers of mesothelial origin, such as calretinin (A) and CK 5/6(+) (B).** 10× magnification.

The preoperative abdominal CT scan demonstrated a well-circumscribed, solid right adrenal mass measuring 88 × 80 × 90 mm. Based on the above findings, the primary diagnosis was non-functional adrenal carcinoma. Subsequently, an open total left adrenalectomy was performed and no lymphadenopathy was seen. On gross examination the tumor was a well-circumscribed solid mass. The histological appearance, together with the immunophenotype of this tumor, was consistent with the diagnosis of cystic lymphangioma-like AT of the adrenal gland (Figures 8 and 9).

## Discussion

The adrenal glands are an extremely rare site of occurrence for an adrenal medullary hyperplasia, adenomatoid tumor, hydatid disease or primary adrenal angiosarcoma. Unusual adrenal tumors are frequently misdiagnosed as primary or metastatic malignant enlargement.

AMH as a cause of hypertension and/or palpitations resembling pheochromocytoma is rare, and has been described in patients with multiple endocrine neoplasia (MEN). AMH, when present in patients with familial MEN type 2 (MEN2), usually occurs bilaterally. The occurrence of unilateral AMH not associated with MEN is extremely rare and has been documented only in several cases [3]. This hyperplasia is considered to precede pheochromocytoma in patients with MEN2 [4]. Carney defined a pheochromocytoma as a medullary nodule of 1 cm or larger [3–5]. The diagnosis of adrenal medullary hyperplasia is based on the following criteria: a history of episodic attacks of hypertension and/or tachycardia suggesting pheochromocytoma, combined with increased secretion of catecholamines; diffuse expansion of medulla in the adrenal gland with or without nodular formation; adrenal medulla composed of enlarged cells with or without pleomorphism; and an adrenal cortico-medullary ratio of less than 10:1 [3].

The patients described in this study had AMH without evidence of MEN2 or a family history of pheochromocytoma. A pathologic study showed diffuse adrenal medullary hyperplasia that decreased the cortico-medullary ratio. Because of the well-recognized metachronous nature of the adrenomedullary lesions, patients will maintain prolonged follow-up after successful unilateral adrenalectomy [3, 4, 6]. Normalization of catecholamines excretion and the lack of clinical sings (three (case one) and seven (case two) years following unilateral adrenalectomy) excluded bilateral AMH. Similar good results with conservative surgery in nonhereditary AMH have been reported by other authors [7].

Hydatid disease (Echinococcosis) is a parasitic infection caused by several species of the cestode Echinococcus. The most common form is *Echinococcus granulosus*; much less common is *Echinococcus multilocularis*. Hydatid cysts occur throughout the world but are endemic in the pastoral and farming regions of the Mediterranean, Eastern Europe, the Middle East, South America, Australia and South Africa [8, 9].

Hydatid cysts may be found in almost any part of the body, but most often they are found in the liver and lungs. A hydatid cyst of adrenal gland is very rare; this entity is found in only 7% of all adrenal cysts. Parasitic cysts involving the adrenals are usually secondary and are part of generalized Echinococcosis. Rarely is Echinococcus infection limited to the adrenal gland. Only a few cases of primary hydatid cyst of the adrenal gland have been reported [8]. Our patient (case three) had a primary hydatid cyst of the right adrenal gland.

Since most adrenal cysts are asymptomatic, they are usually incidental findings on imaging studies or discovered incidentally during surgery performed for other abdominal pathologies. The sensitivity of a CT scan in an abdominal hydatid cyst is 97%. To identify a hydatid cyst, CT and/or magnetic resonance imaging can reveal a cystic lesion and the presence of daughter cysts [8, 9]. On CT scans, concentric areas of separation and calcification indicate that a cyst is of parasitic origin. The most common presenting symptom is pain, accompanied by intra-cystic hemorrhage, rupture or infection. Anaphylactic shock may be caused by the rupture of hydatid cysts [9]. Our patient (case three) was completely asymptomatic and the adrenal tumor was detected incidentally. Hydatid disease of the adrenal gland with hormonal function has not been reported. Eosinophilia occurs in one fourth of cases and was present in our patient (case three). There are many sensitive and specific serological tests available, such as complement fixation, enzyme-linked immunosorbent assay, ARC 5 (activity regulated cytoskeleton) precipitation and specific hydatid IgE (Immunoglobulin E) tests. The sensitivity of serologic tests is 90% [8, 9]. We did not use specific tests because hydatid disease was not suspected.

The definitive treatment method for hydatid cysts of the adrenal gland is surgical excision [9]. We qualified our patient (case three) for surgery because of the malignancy risk (size greater than 4 cm) [1, 2], and the patient underwent laparoscopic total adrenalectomy. Most authors of previous reports performed adrenalectomy because of the destruction of the organs by a large cyst, but El Idrissi Dafali performed a simple cystectomy [8, 10]. When peritoneal spillage is suspected, and when the patient has a coexistent peritoneal cyst, antihelmintic drugs are recommended [8].

PAA is an extremely rare aggressive neoplasm. The first example of PAA was described by Kareti in 1988 [11–14]. Patients usually present non-specific symptoms like abdominal pain. In most cases the lesion is over 6 cm [12]. On macroscopic examination, the tumor suggests pheochromocytoma or adrenal carcinoma. On histopathological examination, PAA displays epithelioid differentiation with large, rounded neoplastic cells with vesicular nuclei and prominent nucleoli. On immunohistochemical analysis, the angiosarcoma component expresses endothelial markers CD31, CD34 and, less extensively, von Willebrand factor (factor VIII) [12, 13].

Angiosarcoma is a local invasive neoplasm. An open adrenalectomy and block resection approach would have been chosen for patients suspected of having PAA. This tumor is derived from vascular endothelial cells, therefore the traditional chemotherapy agents ifosfamide and epirubicin were used in combination with anti- angiogenic drugs bevacizumab or sorafenib. The chemotherapy plan for patient four was doxorubicin and ifosfamide but she refused the treatment. Bevacizumab is effective for angiosarcomas in 57% patients. Sorafenib provides a better progression-free survival, but the response rate is lower than standard cytotoxic agents. Radiation therapy may be not effective [12]. In the largest series of nine PAA tumors found in literature, thee patients survived, three died from the recurrence of the disease and three died of any other causes [13, 14]. Our patient (case four) had recurrence of the disease and died after two years.

AT is a rare benign neoplasm of mesothelial origin which usually occurs in the genital tract of both sexes. Occasionally these tumors are found in other locations such as the heart, pancreas, skin, pleura, omentum, lymph nodes, retroperitoneum, intestinal mesentery and adrenal glands. So far in the English literature only 27 cases of adrenal AT have been described [15]. Most patients with the adrenal gland AT were men; only one tumor was reported in a woman. A possible explanation for this disproportion can be connected with the differences in embryological development of the gonads in both sexes, especially the different role of the mesonephric ducts in males and females [15, 16].

ATs are usually asymptomatic. In the available literature only one patient had symptoms which could be connected to the adrenal mass. In our patient (case five) the adrenal tumor was found incidentally.

The adrenal AT tumor can mimic all other entities occurring in this area such as adenomas, carcinomas, pheochromocytomas, myelolipomas, benign cystic lesions and metastases from distant locations. It may also be easily misdiagnosed as a vascular neoplasm (lymphangioma or angiosarcoma). The immunohistochemical profile was consistent with the one described in AT in usual locations (genital tracts) and was typical for a tumor of mesothelial origin: calretinin (+), CK(cytokeratin) 5/6 (+), CK 7(+), vimentin (+), D2-40 (+), CD31(-) and CD34 (-).

Numerous irregular cystic spaces lined with flattened cells may suggest a vascular neoplasm, especially lymphangioma. However, the cells are positive for CK and they lack reactivity to the usual vascular markers CD31, CD34, factor VIII, which exclude vascular tumors. It is important that AT may show reactivity to D2-40, a marker which is known to be positive in the endothelial cells of lymphatic vessels. In at least two cases reported in the literature, AT were primary misdiagnosed as lymphangioma cavernosum [16–18] similar to our case five.

Radiology has the important role in the detection of adrenal masses. CT is usually the primary imaging modality for the differentiation of adrenal masses. A large tumor diameter, unclear boundaries, infiltration of the neighboring structures, non-homogenous density and the presence of a thick, irregular peripheral zone becoming enhanced after contrast administration may suggest a malignant character of the lesion, however the specificity of those criteria does not exceed 75% [1, 2]. Most centers recommend excision of a lesion sized 4 cm or more, as we advised in our department.

A medical history and physical examination may suggest possible cause. For example, paroxysmal hypertension may suggest a hormonally active mass, like in our AMH cases. Evaluation of hormonal excess should be done when imaging characteristics do not univocally suggest a necessity for surgery.

The clinician and surgeon must make the decision to operate jointly, trying to achieve the best calculus of what is known and predicted. Finally, histopathological examinations could be surprising.

## Conclusions

In the differentiation of adrenal tumors, we should first take into consideration benign lesions, followed by malignancies, metastases and pheochromocytomas.

The suggestion of malignancy in CT scans was the reason for total adrenalectomy in three our cases.

Hormonal activity inclined us to laparoscopic adrenalectomy in two cases.

Finally, in all cases the final histopathological reports were surprising and unexpected.

## Consent

Written informed consent was obtained from the patients for publication of this report and any accompanying images.

## Abbreviations

AMH: Drenal medullary hyperplasia
ARC: Activity regulated cytoskeleton
AT: Adenomatoid tumor
CD: Cluster of differentiation
CK: Cytokeratin
CT: Computer tomography
IgE: Immunoglobulin E
MEN: Multiple endocrine neoplasia
PAA: Primary adrenal angiosarcoma
USG: Ultrasonography.
